# Anticancer Potential of Post-Fermentation Media and Cell Extracts of Probiotic Strains: An In Vitro Study

**DOI:** 10.3390/cancers14071853

**Published:** 2022-04-06

**Authors:** Adriana Nowak, Małgorzata Zakłos-Szyda, Justyna Rosicka-Kaczmarek, Ilona Motyl

**Affiliations:** 1 Department of Environmental Biotechnology, Faculty of Biotechnology and Food Sciences, Lodz University of Technology, Wolczanska 171/173, 90-530 Lodz, Poland; ilona.motyl@p.lodz.pl; 2 Institute of Molecular and Industrial Biotechnology, Faculty of Biotechnology and Food Sciences, Lodz University of Technology, Stefanowskiego 2/22, 90-537 Lodz, Poland; malgorzata.zaklos-szyda@p.lodz.pl; 3 Institute of Food Technology and Analysis, Faculty of Biotechnology and Food Sciences, Lodz University of Technology, Stefanowskiego 2/22, 90-537 Lodz, Poland; justyna.rosicka-kaczmarek@p.lodz.pl

**Keywords:** probiotics, postbiotics, lactic acid bacteria, cancer, Caco-2, anticancer activity, reactive oxygen species, mitochondrial membrane potential, apoptosis

## Abstract

**Simple Summary:**

Probiotics and their metabolites are very important for human health. The aim of this research was to determine probiotic strains with the strongest inhibitory properties against intestinal cancer cells. As a result of the screening, it was possible to find two strains, i.e., *Lactiplantibacillus plantarum* 0991 and *Levilactobacillus brevis* 0983, that could inhibit the proliferation of cancer cells by induction of oxidative stress and programmed cell death. Both strains exhibit interesting anticancer properties and potential as functional food ingredients; however, the results must be confirmed in further research.

**Abstract:**

Background: Lactic acid bacteria (LAB), many of which are probiotics, can produce health-promoting metabolites (postbiotics). Purpose: To assess the mechanism of antiproliferative action of postbiotics, post-fermentation media (PFM) and cell extracts (CEs) of several strains of LAB were studied against colon (Caco-2), and cervix (HeLa) cancer cell lines, as well as normal intestine (IEC-6) cells, were used as a comparison. Methods: Postbiotics of various LAB (*n* = 39) were screened for their antiproliferative activity. The effect of PFM and CEs on reactive oxygen species (ROS), mitochondrial membrane potential (MMP), ATP production, phosphatidylserine (PS) externalisation, and apoptosis-related caspases 3/7 and 9 activation was assayed. Results: PFM and CEs showed strong dose-dependent antiproliferative activity against Caco-2 cells, up to 77.8 ± 0.8% and 58.4 ± 1.6% for PFM and CEs, respectively. Stronger inhibitory activity against cancerous (Caco-2 and HeLa) cells than against normal (IEC-6) cells was observed. PFM were more inhibitory than CEs, and both generated oxidative stress in Caco-2 cells. PFM of *L. plantarum* 0991 and *L. brevis* 0983 induced apoptosis in Caco-2 cells by the mitochondrial signalling pathway. Conclusions: Anticancer activity of PFM and CEs of LAB, as well as the ability of apoptosis induction, is strain-specific.

## 1. Introduction

According to reports by the World Health Organisation (WHO), cancer is one of the leading causes of death worldwide, and the main reasons for cancer are smoking, infections, improper diet, and the so-called Western-type lifestyle [1]. Malignancies typical for affluent societies are, inter alia, cancers of the colon/rectum and uterus (endometrial carcinoma) [1]. Colorectal cancer (CRC) is the third most common cancer in the world, with 940,000 new cases annually, of which 500,000 people die each year [1]. Colon cancer mortality is predicted to almost double in the next 20 years [2].

The human gastrointestinal tract (GIT) inhabits more than 10^14^ cells of microorganisms that establish the human microbiome which affects the body both during homeostasis and disease [3]. The microbiota regulates many physiological processes responsible for metabolism, neutralisation of toxins, immune regulation, and resistance to pathogens. As a result, they maintain homeostasis in the host’s body and ensure health [3]. The composition of the gut microbiota can be regulated by dietary components such as probiotics that increase the number of lactic acid bacteria (LAB), which may, in turn, impact the carcinogenesis process associated with colon cancer [4]. Therefore, many studies are currently conducted to study the mechanism and effectiveness of probiotics and postbiotics on cancer cell lines. The widely accepted scientific definition of probiotics around the world is ‘Live microorganisms that, when administered in adequate amounts, confer a health benefit on the host’ [5]. In 2021, the experts’ panel also clarified the definition of postbiotics, which is ‘a preparation of inanimate microorganisms and/or their components that confers a health benefit on the host’; thus, a postbiotic must include some non-living microbial biomass, whether it can be whole microbial cells or cell constituents and fragments (cell walls, membranes, exopolysaccharides, cell wall anchored proteins, pili, etc.) or a mixture of post-fermentation metabolites (organic acids, peptides, secreted proteins, enzymes, bacteriocins, hydrogen peroxide, vitamins, etc.) [6].

Since probiotics and their postbiotics can modify the intestinal microbiota, e.g., by increasing the number of lactobacilli and bifidobacteria, they influence the process of colon carcinogenesis. This is possible through several mechanisms, which were discussed in detail in our previous review [7] and examples of which include the following [4,7]:

(a) Antiproliferative activity—according to many studies, both probiotics and postbiotics demonstrate antiproliferative activity against cancer cell lines [8,9,10,11,12,13,14]. It can manifest through the activity of mitochondrial dehydrogenases. Most likely, the action is based on the cell cycle arrest in the G1 phase, which blocks the cell’s transition to the next phase of the cycle (S), and it is impossible for replication and cell division to occur. Carcinogenesis is arrested in the promotion phase, and the tumour does not continue to grow [15];
(b) Induction of cancer cell apoptosis, or genetically programmed cell death—a mechanism used to control the number of cells in a multicellular organism. During the development of neoplastic tissue, the altered cells become resistant to signals directing them down the path of apoptosis. Research shows that probiotic bacteria and postbiotics may play major roles in regulating the internal and external pathway of apoptosis, which may be a key defence mechanism against colon cancer [10,14,16,17,18,19];
(c) Production of compounds with a cytoprotective effect (antiproliferative and proapoptotic) for the intestinal epithelial cells such as organic acids including short-chain fatty acids (SCFAs) and lactic acid (LA). They can display antiproliferative activity and induce apoptosis in cancer cells [20].

In our previous in vitro studies, we demonstrated that LAB strains can modulate GIT microbiota by decreasing the activity of bacterial enzymes, including *β*-glucuronidase involved in xenobiotic metabolism; reducing carcinogens level and their detoxification; protecting colon cells against DNA damage, and promoting DNA repairs [21,22,23].

The aim of this current research is to demonstrate the mechanism of antiproliferative and cytotoxic activity of post-fermentation media (PFM) and cell extracts (CEs) of several strains of LAB, including commercial probiotics and potential probiotics. In the first step, the antiproliferative activity of PFM and CEs of 39 LAB strains against human adenocarcinoma cell line Caco-2 cells was screened. Based on the obtained results, PFM and CEs of five strains with the strongest antiproliferative activity were selected. Then, their suppressive activity was tested on two other cell lines—human cervix adenocarcinoma HeLa and rat normal small intestine IEC-6. Then, the assays for selected PFM and CEs were narrowed to only Caco-2 cells, and the biological activities of Caco-2 cells treated with PFM and CEs were examined with the application of various cellular and morphological methods regarding metabolic activity and antiproliferation studies, mitochondrial membrane potential (MMP), intracellular oxidative stress generation, and apoptosis induction (morphological and biochemical observations). To the authors’ knowledge and based on their review of the literature data, the current article presents the results of screening the antiproliferative activity of PFM and CEs among a great number of genera, species, and strains of LAB, and some species such as *Amylolactobacillus amylophilus*, *Secundilactobacillus similis*, *Lentilactobacillus diolivorans*, or *Limosilactobacillus mucosae* that have not been studied so far. The ability of PFM and CEs to generate hydrogen peroxide alone in cancer cells, as well as the level of phosphatidylserine (PS), externalised on the outer leaflet of the cell membrane of apoptotic cells, has not yet been investigated.

## 2. Materials and Methods

### 2.1. Chemicals, Culture Vessels, and Other Materials

Phosphate buffer saline (PBS), deMan–Rogosa–Sharp (MRS) broth, glass beads, high-glucose and low-glucose DMEM, RPMI 1640, 4-(2-hydroxyethyl)-1-piperazineethanesulphonic acid (HEPES), streptomycin–penicillin mixture for cell cultures, insulin, 3-(4,5-dimethylthiazol-2-yl)-2,5-diphenyltetrazolium bromide (MTT), 4′,6-diamidino-2-phenylindole (DAPI), 2′,7′-dichlorofluorescin diacetate (DCFH–DA), dimethyl sulphoxide (DMSO), paraformaldehyde, hydrogen peroxide (H_2_O_2_), trypan blue, cyanide m-chlorophenylhydrazone (CCCP), Fluorometric Hydrogen Peroxide Assay Kit (MAK 165), acids (lactic, butyric, acetic and propionic), Annexin-V-FITC Assay Kit, propidium iodide (PI) and 0.22 µm pore size filters were purchased from Merck Life Science, Warsaw, Poland. Foetal bovine serum (FBS), GlutaMAX™, TrypLE™ Express, tetraethylbenzimidazolylcarbocyanine iodide (JC-1), roux flasks T75 were purchased from Thermo Fisher Scientific, Waltham, MA, USA. Cryobanks™ were from Copan Diagnostics Inc., Jefferson Avenue Murrieta, Murrieta, CA, USA. CellTiter-Glo^®^ Luminescent Cell Viability Assay, Caspase-Glo^®^ 3/7 Assay, and Caspase-Glo^®^ 9 Assay were purchased from Promega Corp., Madison, WI, USA. Caco-2 cells were from Cell Line Service GmbH, Eppelheim, Germany, while IEC-6 cells were purchased from DSMZ German Collection of Microorganisms and Cell Cultures GmbH, Braunschweig, Germany. In addition, 6-, 24- and 96-well plates (Greiner Bio-One GmbH, Kremsmünster, Austria) were used, and 4- and 8-well IBIDI LabTek II CC2 chambered coverslips were purchased in Biokom Systems, Janki, Poland.

### 2.2. Bacterial Strains: Culture, Propagation, Freezing, and Storage

For the initial screening of antiproliferative activity, 39 strains of LAB (belonging to 10 genera and 14 species) were tested. These were *Lactiplantibacillus plantarum* (0981, 0982, 0989, 0990, 0991, 0995, 0996); *Levilactobacillus brevis* (0983, 0984, 0912, 0950); *Lacticaseibacillus paracasei* (0985, 0913, 0993); *Lactobacillus delbrueckii* (0851, 0987); *Leuconostoc mesenteroides* (0986, 0994); *Lacticaseibacillus rhamnosus* (0900, 0902, 0908, 0997, GG); *Limosilactobacillus mucosae* (0988); *Lacticaseibacillus casei* (0903, 0919, 0924, DN 114-001, Shirota), *Lactobacillus acidophilus* (0839, 0925, 0937, 0946); *Lactobacillus helveticus* (Z/1); *Secundilactobacillus similis* (04/2); *Lentilactobacillus diolivorans* (04/1); *Pediococcus parvulus* (4/2K, 02/1); *Amylolactobacillus amylophilus* (0843) and additionally commercial probiotic non-lactic acid bacterium *Bifidobacterium breve animalis* ssp. *lactis* Bb-12. The strains, depending on the species, were isolated from homemade fermented foods (cucumbers, sauerkraut, sourdough, soured milk) or infant and human faeces. Commercial probiotic strains were either gifts or were isolated from probiotic products available on the market.

The strains of LAB were acquired from the own collection of the Department of Environmental Biotechnology and from the Pure Culture Collection of the Institute of Fermentation Technology and Microbiology (LOCK 105), Lodz University of Technology. Some commercial probiotic strains were also used in research: *L. rhamnosus* 0900 and 0908 and *L. casei* 0919 are applied to the production of Latopic preparation (Biomed, Poland) recommended for children with atopic dermatitis [24], and *L. casei* DN 114-001 and Shirota are present in probiotic yogurts, while *L. rhamnosus* GG is sold in Dicoflor probiotic preparation.

The strains were stored in Cryobanks™ at −20 °C. Before experiments, they were activated and threefold passaged (3% inoculum) in MRS broth for 24 h in anaerobic conditions (5% CO_2_), at 30 or 37 °C (depending on the species/origin).

### 2.3. PFM and CE Preparation

PFM were prepared as follows: the liquid MRS medium was inoculated with an individual strain of bacteria and then incubated for 24 h at the appropriate temperature. The samples were centrifuged (10,733× *g*, 15 min); then, the pH of the supernatants was adjusted to 7.0 ± 0.1 (with 0.1 M NaOH and HCl) to eliminate the cytotoxic effect of acidic pH on cells. Next, the supernatants were filtered using sterile syringe filters (0.22 μm) and frozen in test tubes until analysis at −20 °C. In order to prepare CEs, PBS (pH 7.2) was added to the remaining pellets. After thorough mixing with glass beads, samples thus prepared were disintegrated (5 min, amplitude 50, pulse 6 s, pause 2 s, ice bath) with an ultrasonic homogeniser (Hielscher Ultrasonics GmbH, Germany). Then, CEs were centrifuged (10,733× *g*, 15 min), supernatants pH was adjusted to 7.0 ± 0.1, filtered (0.22 μm), and frozen at −20 °C until analysis.

### 2.4. Cell Cultures (Caco-2, HeLa, and IEC-6)

Caco-2 (human colon adenocarcinoma) and HeLa (human cervical adenocarcinoma) were cultured in high-glucose DMEM, while IEC-6 (normal small intestine from rat) in low-glucose DMEM:RPMI 1640 (1:1, *v/v*), with the addition of 10% FBS, 4 mM (Caco-2), or 2 mM (IEC-6) GlutaMAX™, 25 mM HEPES, 100 µg/mL streptomycin/100 IU/mL penicillin, and 0.1 U/mL insulin (IEC-6). Cells were incubated at 37 °C, with 5% CO_2_, in a humidified incubator for 7 days, to reach 80% confluence. Every three days, the cells were washed with PBS (pH 7.2), and the medium was renewed. Confluent cells were detached from the culture with TrypLE™ Express (37 °C, 6–12 min), centrifuged (307× *g*, 5 min), and decanted, and then the pellet was re-suspended in a fresh culture medium. After performing a cell count by haemacytometer and determining cell viability by trypan blue exclusion, the cells were ready to use. Caco-2 epithelial cells are the most common in vitro cell-based research model regarding probiotic–LAB interactions and the human gastrointestinal tract because, in culture, they display morphological, structural, and functional properties similar to those of intestinal enterocytes.

### 2.5. Antiproliferation Assays (MTT)

#### 2.5.1. PFM and CEs of 39 LAB Strains against Caco-2 Cells—Strain Screening

The final tested concentrations of PFM and CEs of LAB strains were 1%, 5%, 10%, and 20% (*v/v*). The assay was conducted as described previously [25]. In short, 10,000 Caco-2 cells were seeded in each well of a 96-well plate in a complete culture medium. The cells were incubated overnight at 37 °C under 5% CO_2_, and the medium was aspirated; then, PFM or CEs were added to each well in four repeats. The vehicle (negative control) contained cells in a culture medium. Cells were exposed to PFM and CEs for 48 h (37 °C, 5% CO_2_); next, test samples were aspirated from above the cell monolayer from each well, and MTT (0.5 mg/mL) was added and incubated for a further 3 h. Then, MTT was removed, and formazan precipitates were solubilised with DMSO. Absorbance was measured at 550 nm with a reference filter of 620 nm, using a microplate reader (TriStar^2^ LB 942, Berthold Technologies GmbH & Co. KG, Bad Wildbad, Germany).

#### 2.5.2. PFM and CEs of Selected LAB against HeLa and Normal IEC-6 Cells

The cytotoxic activity of PFM and CEs against HeLa and IEC-6 cells was conducted (as described in Section 2.5.1) only for five selected strains with the strongest antiproliferative effect against Caco-2 cells.

#### 2.5.3. Pure LA and SCFAs against Caco-2 Cells

The acids (lactic, butyric, acetic, and propionic) were diluted in culture DMEM to receive the final tested concentrations as follows: 0.1; 0.2; 0.4; 0.8; 1.6; 3.1; 6.3; 1.3; 2.5, and 5.0 mg/mL. Then, the samples were added to the monolayers of Caco-2 cells in a 96-well plate and the cells were exposed for 48 h at 37 °C under 5% CO_2_. Then, the MTT assay was performed as described in Section 2.5.1. The IC_50_ was calculated according to OECD protocol [26].

### 2.6. Clonogenic Assay

To each well of a 6-well plate, 50,000 cells were seeded and cultured to reach 80% confluence. After that, cells were washed with PBS and exposed to PFM and CEs of selected LAB for 60 min. The positive control was 50 µM H_2_O_2_. All cells in each well were harvested and counted in a haemocytometer. Next, 1000 cells were inoculated in each well of the 6-well plate and cultured for 7 days to enable the formation of the colonies. The colonies were fixed with 3.7% paraformaldehyde for 15 min, air-dried, and stained with 0.1% crystal violet. The morphology of Caco-2 cells monolayer was observed under 5× objective in an inverted microscope Nikon Ts2 with EMBOSS contrast (Nikon, Tokyo, Japan) and a Jenoptic Subra Full HD Colour digital camera (Jena, Germany).

### 2.7. ROS Generation and H_2_O_2_ Level Quantification

For the experiment, 10,000 Caco-2 cells were seeded in each well of a 96-well black plate in a complete culture medium. The cells were exposed to PFM and CEs of selected LAB, as described in Section 2.5.1. After the exposition, PFM and CEs were aspirated, cells were washed with PBS, and 20 µM DCFH–DA was added to each well with culture media without FBS and incubated for 40 min (37 °C, 5% CO_2_). The negative controls contained cells in DMEM (without FBS), while positive controls contained cells in DMEM (without FBS) with H_2_O_2_ (200 mM). After incubation, the fluorescence was measured (excitation/emission = 490/530 nm). The average DCF fluorescence was determined as a percentage (%) relative to the negative control, which was assumed to be 100%. For microscopic observations, Caco-2 cells were cultured in 8-well LabTek II CC2 chambered coverslips in the amount of 50,000/well. The intracellular fluorescence of cells was observed under a fluorescent microscope (Nikon Eclipse Ci H600L, Japan) attached to a digital camera (Nikon Digital Sight DS-U3) and imaging software (NIS-elements BR 3.0, Nikon) with a 20× objective. Increased intensity of intracellular fluorescence was indicative of an increased level of generated ROS. Assay for H_2_O_2_ level quantification was conducted with the application of Fluorometric Hydrogen Peroxide Assay Kit, according to the manufacturer’s instruction. Firstly, the standard curve of the H_2_O_2_ dose–response was prepared. The H_2_O_2_ concentration for the samples was determined from the above curve. Positive control wells (cells treated with 10 mM H_2_O_2_) and negative control wells (cells only and medium only) were used.

### 2.8. Mitochondrial Membrane Potential (MMP) Measurement

For the experiment, 10,000 Caco-2 cells were seeded in each well of a 96-well black plate in a complete culture medium. The cells were exposed to PFM and CEs of selected LAB, as described in Section 2.5.1. The negative control contained cells in DMEM, while the positive control contained cells in DMEM with CCCP (50 µM). After the exposition, the medium with test samples was aspirated, and JC-1 dye in DMEM without FBS at the final concentration of 1 µg/mL was added to each well and incubated for 20 min (37 °C, 5% CO_2_). After incubation, the fluorescence was measured (excitation/emission = 490/530 nm). For microscopic observations, Caco-2 cells were cultured in 4-well LabTek II CC2 chambered coverslips in the amount of 100,000/well. The intracellular fluorescence of cells was observed under a fluorescent microscope with 10× and 20× objectives.

### 2.9. Measurement of ATP Production

Intracellular ATP level was determined with CellTiter-Glo^®^ Luminescent Cell Viability Assay. After cells incubation with the PFM or CEs, the single reagent was added directly to the cells, and a luminescent signal proportional to the amount of present ATP was generated and measured (BioTek, Winooski, VT, USA).

### 2.10. PS Externalisation and Membrane Permeabilisation

To quantify the level of PS externalised on the outer leaflet of the cell membrane of apoptotic cells, an Annexin-V-FITC Assay Kit was used. After treatment, cells were washed twice with PBS and incubated with annexin-V-FITC (at a final concentration of 0.25 μg/mL) for 20 min. Annexin-V binding was measured by the change in fluorescence (excitation/emission = 485/530 nm). Membrane permeabilisation caused by investigated samples was measured with PI. After 24 h treatment of cells with the PFM or CEs, PI was added at a final concentration of 1 µg/mL. Intercalation was monitored by the change in fluorescence (excitation/emission = 535/620 nm).

### 2.11. Detection of Caspases 3/7 and Caspase 9 Activity

The late stage of apoptosis was measured with Caspase-Glo^®^ 3/7 Assay or Caspase-Glo^®^ 9 Assay, according to the manufacturer’s instructions. After cells treatment with the PFM or CEs, the appropriate single reagent was added directly to the cells, and a luminescent signal was measured.

### 2.12. Morphology of Caco-2 Cells

#### 2.12.1. Crystal Violet Staining

Morphological changes of Caco-2 cells after exposure to PFM and CEs of selected LAB were observed in 4-well LabTek II CC2 chambered coverslips. Caco-2 cells were seeded on each well by adding 100,000 cells/well, each sample in two repeats. After the exposition, the medium was aspirated, and cells were washed with PBS and fixed with 3.7% paraformaldehyde for 15 min at ambient temperature. The cells were then stained with 0.1% crystal violet. After staining, the wells were washed with PBS until no colour remained and then were air-dried. The morphology of Caco-2 cells was observed at 20× and 40× objectives under an inverted microscope.

#### 2.12.2. DAPI Staining

The nuclear changes in Caco-2 cells in the presence of PFM and CEs were observed using 8-well LabTek II CC2 chambered coverslips. DAPI staining was performed according to the procedure described in Section 2.12.1, each in two repeats. After air-drying, the cells were stained with 1 µg/mL DAPI in the dark. The morphology of cells was observed at 20× objective under a fluorescent microscope.

### 2.13. LA and SCFA Quantification

The quantification of LA and SCFA profiles in PFM was performed according to the method presented by Chen et al. [27], with some modifications. Determination of lactic, acetic, propionic, and butyric acids was conducted using high-performance liquid chromatography (HPLC). LA and SCFAs were analysed qualitatively and quantitatively by comparing standard solutions with the PFM of a selected LAB. The samples were centrifuged (1774× *g*, 10 min), the supernatant decanted and filtered through a 0.22 µm nylon syringe filter into an autosampler vial, and subjected to chromatographic analysis.

The chromatographic separation was determined with a Dionex HPLC + Ultimata 3000 chromatograph, coupled with a UV–Vis detector and an A11606 C18 column (2.1 × 150 mm, particle size 2.6 µm; ATC, Waltham, MA, USA) thermostated at 30 °C, with 10 µL injection volume. Gradient elution was used comprising phosphorus buffer (pH 2.34) (phase A) and acetonitrile (phase B), at a flow rate of 0.8 mL/min. The following gradient program was used: 0 min A: 100%; 10.5 min A: 20%; 19.5 min A: 100%. Standard solutions were used to identify SCFAs, and determinations were made by measuring the absorbance at 210 nm. All measurements were made in duplicate. Direct identification was made by the analysis of characteristic retention times for particular acids. Concentrations were established by calculating the area under each peak and a calibration curve for LA and SCFAs.

### 2.14. Statistical Analysis

All data obtained with the use of cell cultures are presented as mean ± SD calculated from at least three independent experiments. Cells in the control sample were exposed only to the vehicle. All obtained results were subjected to statistical analysis using one-way ANOVA analysis, followed by Scheffe’s multiple comparison test or Dunnett’s test performed using GraphPad Prism 6.0 software (GraphPad Software, Inc., La Jolla, CA, US) or OriginPro 6.1 (Northampton, MA, USA) software at the significance level of * *p* ≤ 0.05, ** *p* ≤ 0.01 and/or *** *p* ≤ 0.001.

## 3. Results and Discussion

### 3.1. Antiproliferative Activity of PFM and CEs

The antiproliferative effect of PFM and CEs of all 39 LAB strains against Caco-2 cells was first screened (Table 1). The strains with the strongest antiproliferative activity selected for further studies were bolded. As can be seen from Table 1, cell responses to the PFM and CEs were diversified, depending on the genus of bacteria, and species, and rather strain-specific. For 21 strains (Table 1), a stronger inhibitory effect at all concentrations tested or those higher (i.e., 10% and 20%) against Caco-2 cells was observed for PFM (e.g., *L. plantarum* 0996, *L. brevis* 0912, and 0983, *L. acidophilus* 0937), than for CEs. In general, stronger cytotoxicity of PFM was recorded in the presence of the highest concentration tested (i.e., 20%) than in the presence of the same concentration of CEs (e.g., *L. plantarum* 0991, 0995 and 0996, *L. brevis* 0983 and 0912). The strongest antiproliferative effects for 20% CEs were observed in the case of *L. rhamnosus* GG and *P. parvulus* 4/2K, with values of 58.4% ± 1.6% and 58.3% ± 2.0%, respectively. The inhibitory activities of PFM and CEs generally were dose-dependent; however, in some strains, they were at a comparable level, regardless of the concentration (e.g., *L. rhamnosus* 0908, *L. casei* Shirota, and 0924), while against some strains, weak responses were observed (e.g., *L. rhamnosus* 0997, *L. diolivorans* 04/1). As mentioned above, the antiproliferative activity was rather a strain-specific feature, but all *L. acidophilus* PFM appear to have similarly high activity (in comparison to other species) when exposed to the highest PFM concentration (20%) against Caco-2 cells, and it ranged from 40.4 ± 1.6% (strain 0946) to 58.6 ± 2.1% (strain 0937). *S. similis* 04/2 antiproliferative activity, compared with other strains, was found to be average (Table 1), while *L. diolivorans* 04/1 and *L. mucosae* 0988 showed a weak effect; however, the CEs of *L. mucosae* 0988 appeared to display strong cytotoxicity, ranging from 28.7 ± 2.0 to 44.2 ± 0.6%. PFM and CEs of *A. amylophilus* 0843 were rendered to be one of the most potent inhibitors against Caco-2 cells and were among the top five strains selected for further studies. PFM of the *B. animalis* ssp. lactis Bb-12 displayed a very high antiproliferative activity, ranging from 9.2 ± 0.3% (for 1%) to 59.7 ± 1.2% (for 20%). CEs of this strain were also very cytotoxic (Table 1). *L. mesenteroides* PFM were correspondingly characterised by strong antiproliferative activity, which was a maximum of 68.8 ± 1.6% for strain 0994 and a concentration of 20%. Shukla et al. [28] also proved that bacteria of this genus exhibit anticancer properties. They are supposed to result mainly from the dextran they produce, which has the ability to inhibit the proliferation of cancer cells.

According to many scientific reports, both alive and dead cells of LAB, as well as various cell components, such as the cell wall, peptidoglycan, exopolysaccharides, cytoplasmic fractions, metabolites including SCFAs, conjugated linoleic acids, and bacterial culture supernatants, may show strong antiproliferative effects on neoplastic cells [7,16]. From the literature data, it is evident that intestinal cells are the most common research model on this topic, due to the fact that probiotics and the products of their metabolism (postbiotics) interact with the intestinal epithelium after reaching the intestines. Many authors have investigated the antiproliferative and cytotoxic activity of LAB postbiotics against colon adenocarcinoma cell lines, such as Caco-2, SW-480, CT26, HRT-18, or HT-29 [9,10,11,29,30,31,32,33,34]. The concentrations of the PFM are the most often given as a percentage or mg/mL, less often in µg/mL. The range of the tested concentrations is from 0.47% to 100% [11,35,36,37,38,39] or from 0.0001 to 1000 mg/mL [18,29,31,34,40,41]. In all of the above-mentioned studies, the MTT assay dominates as a tool of antiproliferative activity testing for cancerous cell lines and cytotoxicity assayed to normal cells. In reviewed research, the exposure time of cells to PFM or CEs ranges from 18 to 72 h, and even up to 7 days. Some authors investigate neutralised pH of PFM in the range 7.0–7.4 [9,11,40,42], others investigate the real physiological pH of PFM (e.g., 3.73–6.50) [29,30,35,42], while some research studies do not provide any information. The nomenclature for PFM in the literature is different—e.g., postbiotic metabolites (PMs), cell-free supernatants (CFSs), culture supernatants (CSs), fermentation supernatants (FSs), PFM, cell-free culture supernatants (CFCSs), or ‘bacterial species’ supernatants [11,18,19,35].

In our research, to avoid acidification of the cell culture medium and to eliminate the cytotoxic activity of acidic pH on cell lines, the pH of test samples was neutralised. Our results are in accordance with those reported by Chuah et al. [11], who investigated the antiproliferative effect of PM (pH 7.2–7.4) on 6 strains of *L. plantarum*. They detected a significant inhibitory effect against HT-29 cells after 24–72 h exposition for PM concentrations of 15% and 30% (*v/v*), which was even up to 89%, depending on the strain. The effect was time- and also dose-dependent. Likewise, a strong suppressive dose-dependent effect against HT-29 cells (and against three other cancer cell lines) was observed by Haghshenas et al. [40] after exposition to CFS pH 7.4. The effect was also specific and selective in relation to cell lines. The authors concluded that the protein nature of secreted metabolites of tested strains (Lactococcus lactis ssp. lactis 44Lac and *L. plantarum* 15HN) are responsible for the anticancer action. In one of the studies, the antiproliferative effect of PFM with both physiological and neutral pH was compared. PFM with physiological pH of *Pediococcus* sp. strains showed stronger antiproliferative activity against MCF-7 breast cancer cells than PFM with pH 7.4 [42]. In other studies, a strong dose-dependent decrease in HT-29 cell proliferation was observed by Nouri et al. [35] after 24 h incubation of cells with supernatants (pH 4.2) of *L. rhamnosus* GG. The decrease was more than 80% for the highest concentration tested (25%). It was demonstrated that alive cells of *L. casei* dramatically decreased the viability of colon cancer cells (human HT-29 and murine CT-26) in a time- and dose-dependent manner (after 24 h exposition, a 90% increase in both early and late apoptotic cells was detected), and the effect was stronger if LAB supernatants were only investigated [10]. Authors concluded that the acidic physiological pH of PFM is only partly involved in the antiproliferative effect, which was stronger when alive LAB cells were co-incubated with cancer cells, and viable LAB cells induced the pH decrease, not culture medium alone [10]. *Lactiplantibacillus pentosus* B281- and *L. plantarum* B282-conditioned culture media significantly inhibited proliferation of Caco-2 cells (conc. 25 and 50%), for up to 45%, after 72 h exposition [36]. In our study, PFM inhibited cell proliferation stronger than CEs (Table 1), in contrast to the research of Soltan Dallal et al. [43] in which CEs displayed more powerful antiproliferative activity against Caco-2 cells than LAB supernatants. This activity was also dose-dependent and the strongest for the highest concentrations tested (20%). The antiproliferative effect of PFM of *L. casei* was at a level ranging from 19% (for a concentration of 5%) to 45% (for a concentration of 20%), according to Soltan Dallal et al. [43], which was similar to our strains of *L. casei* tested (i.e., 0903, 0924). Two out of seven isolates of LAB had a good antiproliferative effect against Caco-2 cells after 24 h exposure [31]. For 10% concentration of the PFM of *Limosilactobacillus fermentum*, the viability of cells decreased by 62.8% after 24 h exposition. A concentration level of 1% of CFS of *L. plantarum* A7 and *L. rhamnosus* GG significantly inhibited the proliferation of HT-29 and Caco-2 cell lines (up to 100%) after 48 h exposition [29]. Additionally, heat-killed cells (HKs) of LAB exhibited strong antiproliferative effects. In our study, PFM of *L. rhamnosus* GG was less effective, but CEs showed the strongest antiproliferative action against Caco-2 cells (Table 1). Generally, from our research and the above-mentioned studies, it can be concluded that the neutralised pH of PFM induces a strong inhibitory action at higher concentrations and after a similar or prolonged exposure time than physiological pH. It also depends on the LAB strain being tested and the cell line, as well as on whether PFM, conditioned medium, viable, or inactivated LAB cells were tested.

In our study, five strains (i.e., *L. plantarum* 0991, *L. brevis* 0983, *L. casei* 0919, *P. parvulus* 4/2K, and *A. amylophilus* 0843) belonging to five different genera of LAB were selected for further research. These strains were chosen on the basis of the strongest antiproliferative activity (against the Caco-2 cell line) of both PFM and CEs, but whether a given strain belonged to a different species or genus was also considered. Additionally, the calculation of IC_50_ for PTM for these strains had to be achieved. Next, the antiproliferative activity of PFM and CEs of selected five strains against the cancerous HeLa, and for comparison of the normal intestinal IEC-6 cells was estimated (Table 2). The obtained results show that the inhibitory effect of PFM was generally weak against HeLa cells than against Caco-2, except for the highest tested concentration of PFM, i.e., 20%, which showed to be highly cytotoxic to HeLa cells (above 97% for all strains). CEs did not appear to be, or were only weakly, antiproliferative against HeLa cells, compared with Caco-2. Several studies investigated inhibitory effect of PFM against HeLa [11,17,35,44]. The antiproliferative activity depended on the time of exposition (12–24 h) and concentration (10–50 µg/mL) of PFM or cell lysates—it was time- and dose-dependent [17]. Choi et al. [44] suggested that a strong anticancer agent from *L. acidophilus* is a soluble polysaccharide fraction. Other authors concluded [11] that PM exerts selective inhibitory effects against cancer cells, and it is strain-specific. Again, a clear dose-dependent decrease in HeLa cell viability was determined after 24 h exposure to CS (pH 4.2) of L. rhamnosus GG and *Lactobacillus crispatus* SJ-3C-US [35]. For the highest concentration tested (25%), the decrease in viability was more than 90%.

In our study, PFM showed stronger antiproliferative activity against Caco-2 cells than against IEC-6 at lower concentrations, i.e., 1% and 5%, while higher concentrations (10% and 20%) were strongly cytotoxic against IEC-6 cells, producing up to 99.6 ± 0.1% cytotoxicity in case of all five PFM. CEs always showed more or less strong antiproliferative activity against Caco-2 than IEC-6, for example, *P. parvulus* 4/2K (20% concentration) caused 58.3% ± 2.0% cytotoxicity against Caco-2 and 30.8% ± 0.1% against IEC-6. At concentrations of 5%, 10%, and 20%, PFM and CEs generally showed greater cytotoxic capacity against IEC-6 cells than antiproliferative effect against HeLa.

Many research studies compared the antiproliferative action of PFM/CE/HK against cancerous vs. normal cell lines [11,17,18,29,35,37,40,45,46]. LAB lysates of eight different strains displayed variable inhibitory activity against cancerous vs. normal cell lines [46]. Generally, bacterial lysates definitely showed weaker antiproliferative activity against normal human dermal fibroblasts (HDF) than against cancer cells: Caco-2, MCF-7, HepG-2 (liver), and PC3 (prostate) [46]. This phenomenon depended on both the type of cell line and the bacterial strain. The bacterial lysate, especially of one strain—*Latilactobacillus curvatus* ATCC 51436—showed weaker antiproliferative activity against three out of four tested cancer lines—i.e., against HepG-2 cells than against normal HDF. In our study, PFM in concentrations 10% and 20% were highly cytotoxic to normal IEC-6 cells; however, in lower concentrations (1% and 5%), they were not, or were only weakly, cytotoxic, and they were less cytotoxic than against Caco-2 or HeLa (for HeLa except for 5% PFM of *A. amylophilus* 0843) (Table 2). CEs were always less cytotoxic to IEC-6 than to Caco-2. In some studies, the cytotoxic activity of PFM or CEs on normal cells (e.g., human umbilical vein endothelial cells—HUVEC, glandular epithelium—MCF-10A, liver Chang) was negligible or weak in comparison to cancerous lines (such as HT-29, Caco-2, HeLa, MCF-7, HepG-2, human leukaemia HL60, breast MDA-MB-231, gastric AGS) [11,17,37,40]. However, that is not a rule. Kahouli et al. [18] demonstrated that *L. fermentum* NCIMB 5221 suppressed more Caco-2 cells than normal non-neoplastic colon cells CRL-1831. Human normal lung fibroblasts (MRC-5) were more inhibited than HT-29 and HeLa cells [35]. Normal mouse fibroblasts (L-929) proliferation was inhibited strongly after exposition to CFS of *L. plantarum* A7 and *L. rhamnosus* GG equally with the proliferation of HT-29 and Caco-2 cells [29]. The authors concluded that the antiproliferative activity of the strains is a generic feature of LAB, and it is strain- and cell-line-specific.

In our study, IC_50_ values (the concentration of the test compound required to reduce the cell survival rate to 50% of the control) were counted for the PFM of five selected strains for all three cell lines, and they were determined according to the OECD Guidelines for the Testing of Chemicals (Table 3) [26]. Considering IC_50_ values, the PFM of all strains displayed the greatest antiproliferative activity towards normal intestinal IEC-6 cells. The PFM of *A. amylophilus* 0843 showed the strongest antiproliferative activity towards IEC-6 (5.8%); however, this value was at a similar level as for Caco-2 cells (5.9%). In cancer cells, it appeared that the antiproliferative effect was stronger against Caco-2 than against HeLa for four PFM (except for *L. plantarum* 0991). The least cytotoxic PFM was that of *L. plantarum* 0991 in the case of all three cell lines. The IC_50_ for CEs could only be determined for one strain out of the five selected, i.e., *P. parvulus* 4/2K, at a concentration of 20%, as CEs did not show as strong antiproliferative effects as PFM.

Based on the above results, selected PFM and CE concentrations of the five strains and Caco-2 cells as target cells in human GIT were designated for further study.

### 3.2. Effects of PFM and CEs on Caco-2 Colony Formation

**Figure 1:**
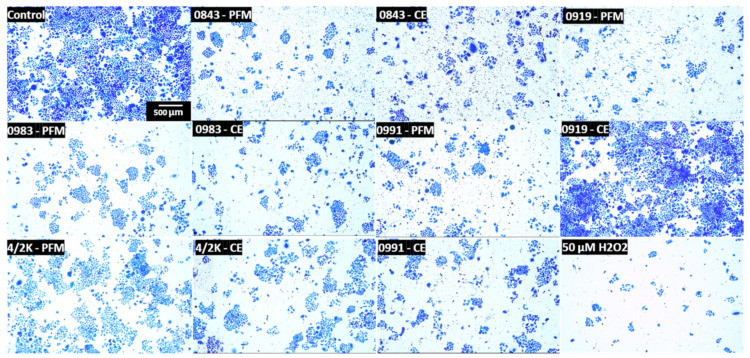
Photographs presenting colonies produced by Caco-2 cells following plating of 1000 cells and 7 days incubation. Cells were treated with 20% (*v/v*) concentration of post-fermentation media (PFM) or cell extracts (CEs) for 60 min. 50 µM H_2_O_2_ was used as a positive control. Negative control, untreated cells. Cells were observed under 5× objective in an inverted microscope Nikon Ts2, with EMBOSS contrast (Nikon, Tokyo, Japan).

After screening 39 initial LAB strains, PMF and CEs of 5 selected strains displayed the strongest cytotoxic and antiproliferative effects against Caco-2 cells. The survival and proliferative capacity of Caco-2 cells treated with PFM and CEs were measured based on their ability to form colonies in a colony-forming assay. Pretreatment of cells with 20% (*v/v*) PFM and CEs effectively inhibited the cell colony formation (Figure 1), which was evidently visible. It was found that the number and size of the colonies formed were significantly decreased after PFM/CE treatment. This confirms that PFM and CEs were able to decrease the metabolic activity (as measured in MTT assay) of Caco-2 cells and also demonstrated antiproliferative effects.

### 3.3. Effects of PFM and CEs on Oxidative Stress and MMP

**Figure 2:**
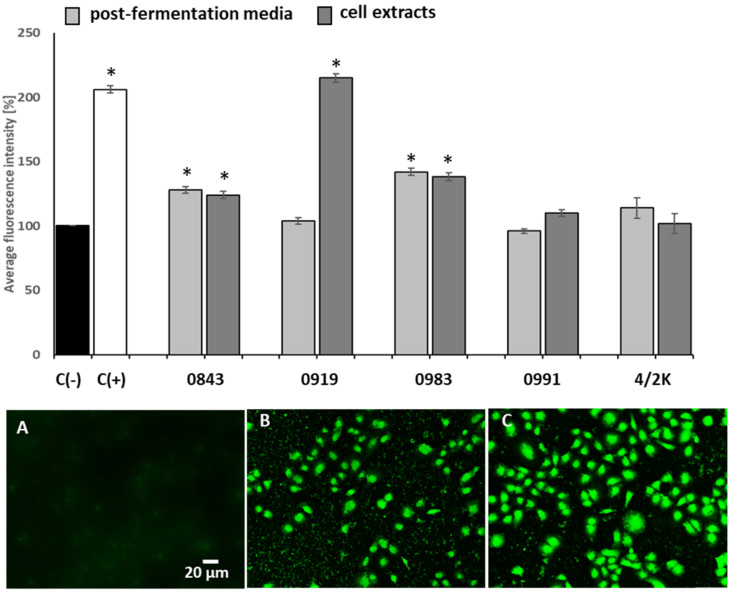
Effect of 5% (*v/v*) post-fermentation media (PFM) and cell extracts (CE) on reactive oxidative species (ROS) generation in Caco-2 cells after 24 h exposure. Each data point represents the mean ± SD, *n* ≥ 4. * Results significantly different from unexposed control cells, C(−), *p* ≤ 0.05: (**A**–**C**) representative microphotographs of 2′,7′-dichlorofluorescin diacetate (DCFH–DA)-stained Caco-2 cells: C(−, untreated Caco-2 cells (**A**); Caco-2 cells exposed to 10% PFM of *L. brevis* 0983 (**B**); C(+), positive control (200 mM H_2_O_2_) (**C**) observed under a fluorescent microscope (Nikon Eclipse Ci H600L, Japan) with 20× objective.

Oxidative stress occurs when the antioxidant balance of cells that are overwhelmed by excess ROS is upset; thus, ROS are regarded as damaging agents, which can lead to apoptosis [47]. In order to study the effect of PFM and CEs on the generation of oxidative stress in Caco-2 cells, 5% (*v/v*) concentrations (not exceeding the IC_50_ values) of PFM and CEs were selected for the experiment, and the cell exposure time was shortened to 24 h. PFM-treated Caco-2 cells resulted in statistically significant (*p* ≤ 0.05) ROS production in the case of two strains: *A. amylophilus* 0843 (128% ± 2.6%) and *L. brevis* 0983 (142% ± 2.8%) (Figure 1). Similar results were observed for CEs of both these strains, and the average DCF fluorescence values of treated cells were 124% ± 2.8% (for *A. amylophilus* 0843) and 138% ± 3.1% (for *L. brevis* 0983) of the vehicle (untreated cells). The most significant generation of ROS (*p* ≤ 0.05) was observed after exposure of cells to CEs of *L. casei* 0919, and the average DCF fluorescence was 215% ± 3.4%, compared with the vehicle (Figure 2). The production of intracellular ROS was analysed by a fluorescence microscope using DCFH–DA, as shown in microphotographs (Figure 2A–C). In treated cells, increased ROS generation by mitochondrial impairment oxidised 2,7-dichlorofluorescein, which, in turn, emitted bright fluorescence.

Hydrogen peroxide is one of the ROS [47]. The level of hydrogen peroxide generated and released by alive cells alone was then quantified after exposure to PFM and CEs (Table 4). The values were read from the standard curve and prepared according to the manufacturer’s instructions. The greatest amount of hydrogen peroxide was released from cells exposed to the CEs of *L. casei* 0919 (18.6 ± 2.7 µM), which is consistent with the general production of ROS (Figure 2). More hydrogen peroxide was released from the cells after exposure to CEs (from 8.8 ± 2.1 to 18.6 ± 2.7 µM) than to PFM (from 2.8 ± 1.9 to 17.1 ± 2.9 µM) (Table 4). The release of hydrogen peroxide for the positive control (10 mM H_2_O_2_) was 26.8 ± 2.9.

**Figure 3:**
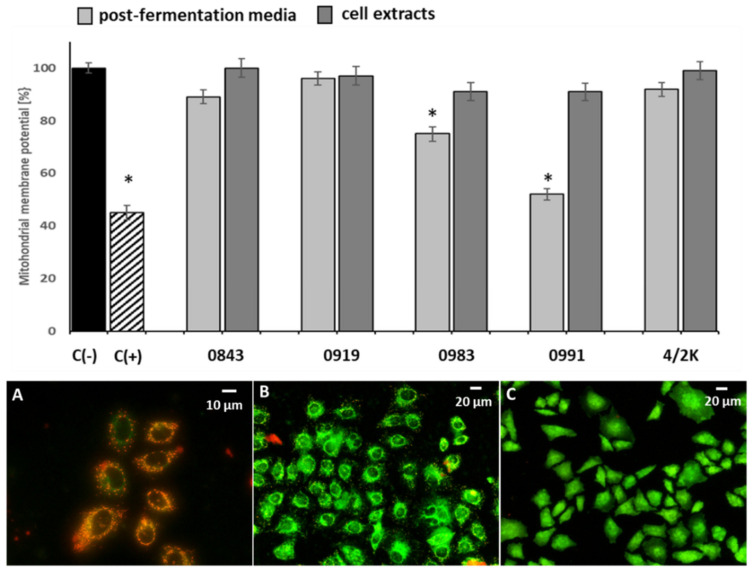
The effect of 5% (*v/v*) post-fermentation media (PFM) and cell extracts (CEs) on mitochondrial membrane potential (MMP) depletion in Caco-2 cells after 24 h exposure. Each data point represents the mean ± SD, *n* ≥ 4. * Results significantly different from unexposed control cells, C(−), *p* ≤ 0.05: (**A**–**C**) representative microphotographs of MMP of Caco-2 cells determined with tetraethylbenzimidazolylcarbocyanine iodide (JC-1) as a fluorescent probe staining method: C(−), untreated Caco-2 cells (**A**); Caco-2 cells exposed to 10% PFM of *L. plantarum* 0991 (**B**); C(+), CCCP, positive control (50 µM) (**C**), observed under a fluorescent microscope (Nikon Eclipse Ci H600L, Japan) with 10× and 20× objectives.

In order to study the effect of PFM and CEs on MMP in Caco-2 cells, 5% (*v/v*) concentration (not exceeding the IC_50_ values) of PFM and CEs was selected, and the time of cells exposure was shortened to 24 h. As presented in Figure 3, a significant (*p* ≤ 0.05) increase in mitochondrial depolarisation was observed only for the PFM of two strains: *L. plantarum* 0991 and *L. brevis* 0983. MMP disruption was the greatest in Caco-2 cells treated with the PFM of *L. plantarum* 0991, in which MMP declined to 51% of the vehicle (untreated cells), while in the positive control (cells treated with 50 µM CCCP), it was 45%. In Figure 3A, Caco-2 cells are presented with high (normal) MMP where JC-1 forms red aggregates. As evident in Figure 3B, Caco-2 cells with MMP depletion exhibit green fluorescence (JC-1 forms monomers), and red aggregates are relatively less frequent, which reveals that the cells can be at the early stages of apoptosis induced by the mitochondrial pathway. In Figure 3C, MMP depletion and green fluorescence are visible.

On the one hand, PFM or CEs of LAB can reduce oxidative stress by scavenging ROS and protecting cells and DNA from damage, and living organisms against cancer and various civilization diseases, which was demonstrated in many studies [7,32,37,44,47,48]. On the other hand, live LAB, PFM, or CEs can induce ROS in cancer cells, leading to their apoptosis [14,19]. Live cells of *Lactobacillus acidophilus* KLDS1.0901 enhanced generation and accumulation of ROS in HT-29 cells after 24 and 48 h exposure, along with the decrease in MMP, leading to apoptosis induced through the mitochondrial pathway [14]. CFCS of *L. casei* SR2, SR2, and *L. paracasei* SR4 (pH 7.3) increased the level of ROS and decreased MMP in HeLa cells after 24 h exposition and in the concentration of 45 µg/mL (which is 0.0045%), which also contributed to apoptosis as evaluated by flow cytometry [19]. Ghoneum and Felo [49] detected that *Lentilactobacillus kefiri* (PFT) lowered MMP and enhanced apoptosis selectively, i.e., in AGF gastric cancer cells, but neither in murine breast cancer 4TI nor in peripheral blood mononuclear cells (PBMCs).

### 3.4. Effects of PFM and CEs on ATP Production, PS Externalisation, Membrane Permeabilisation, and Cell Death

**Figure 4:**
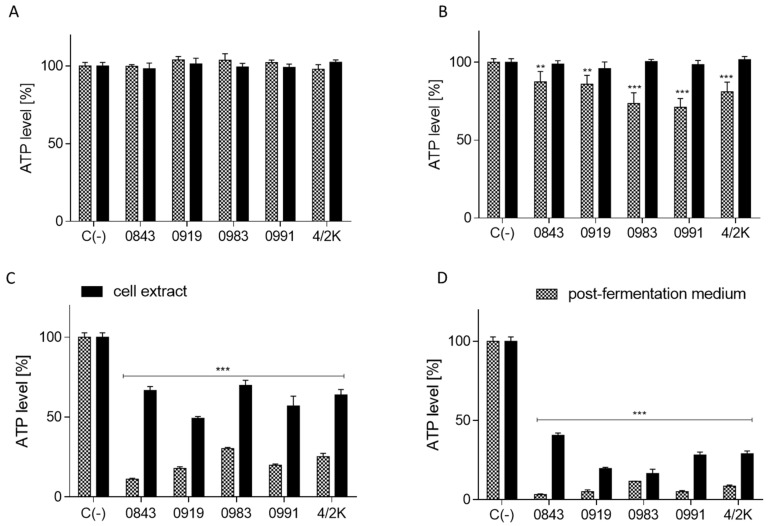
The effect of post-fermentation media (PFM) or cell extracts (CEs) at concentrations (*v/v*) of 0.1% (**A**), 1% (**B**), 10% (**C**), or 20% (**D**) on ATP level in Caco-2 cells after 24 h incubation; values are means ± SD, *n* ≥ 4; statistical significance was calculated versus control cells (untreated), * *p* ≤ 0.05, ** *p* ≤ 0.01, *** *p* ≤ 0.001.

**Figure 5:**
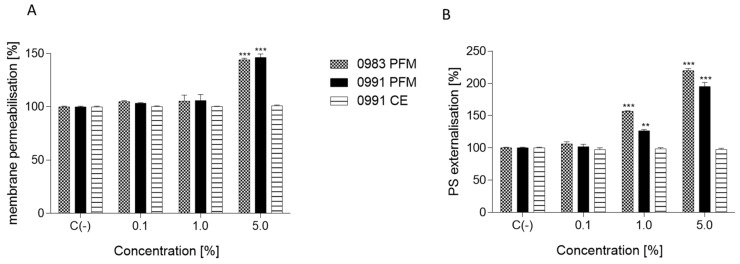
The influence of selected post-fermentation media (PFM) or cell extracts (CEs) on Caco-2 cell membrane permeabilisation detected with propidium iodide (PI) staining (**A**) and phosphatidylserine (PS) externalisation on the outer membrane leaflet of apoptotic cells and detected with Annexin-V-FITC Assay Kit (**B**) after 24 h incubation; values are means ± SD, *n* ≥ 4; statistical significance was calculated versus control cells (untreated), ** *p* ≤ 0.01, *** *p* ≤ 0.001.

**Figure 6:**
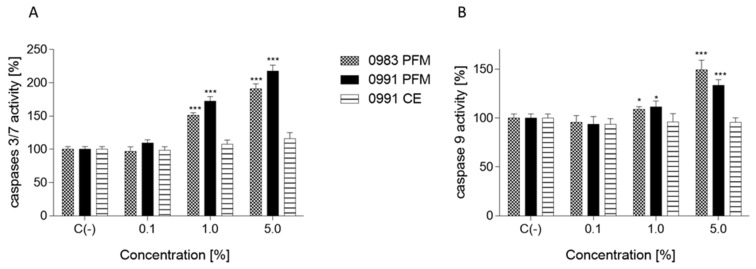
The influence of selected post-fermentation media (PFM) or cell extracts (CEs) on the activation of caspases 3/7 (**A**) and caspase 9 (**B**) in Caco-2 cells after 24 h incubation; values are means ± SD, *n* ≥ 4; statistical significance was calculated versus control cells (untreated), * *p* ≤ 0.05, *** *p* ≤ 0.001.

Since observed PFM and CE cytotoxic activity in Caco-2 cells, the intracellular ATP level was determined after incubation with selected concentrations of PFM and CEs (between 0.1% and 20%, *v/v*) obtained from selected five strains. As presented in Figure 4, only the lowest tested concentration (0.1%) had no influence on ATP generation. The CEs did not change the ATP level in Caco-2 cells within the range of 0.1–1% (Figure 4A,B). However, cells incubation with increased concentrations of CEs strongly influenced Caco-2 energetic level, leading to the depletion of ATP between 60% and 80% in comparison to the control cells. Moreover, all PFM effectively decreased ATP levels in Caco-2 cells by 15–25%, at the concentration of 1%. The strongest effect was observed for PFM of *L. brevis* 0983 and *L. plantarum* 0991. Further experiments showed that treatment of Caco-2 cells with 10% of all studied samples had a high cytotoxic effect, whereas the 20% concentration led to a decrease in ATP to the level 5–40% (Figure 4C,D). Obtained results suggest that cell death in Caco-2 was induced, even after treatment with low concentrations of PFM of *L. brevis* 0983 and *L. plantarum* 0991. Therefore, in the next step, the identification of cell death type was performed for concentrations ranging between 0.1% and 5%. For direct comparison of biological activity of PFM with high cytotoxic potential (*L. brevis* 0983 and *L. plantarum* 0991), selected CEs were obtained from *L. plantarum* 0991. As presented in Figure 5, both PFM at 1% had no effect on membrane permeabilisation (studied with PI staining), whereas they elevated the translocation of PS to the outer leaflet of the cell membrane by 25–50% (determined with Annexin V staining). This suggests the induction of apoptosis, which was further confirmed by the detection of activity of caspases 3/7. Indeed, both PFM at concentrations of 1% increased the activity of caspases 3/7 by almost 50% (Figure 6A). The activation of caspase-9 inhibits DNA repair and induces cytoskeletal disorders, DNA fragmentation, and cell death [50]. Additionally, the observed enhanced activation of caspase 9 (Figure 6B) suggests the mitochondrial signalling pathway of apoptosis, which is in line with the previous results demonstrating ATP depletion and a decrease in MMP. As presented in Figure 6, the elevation of PFM samples concentration to 5% significantly induced membrane permeabilisation and PI accumulation in nuclei, which, along with PS fluorescence enhancement, suggest that these cells are in the late apoptotic stage or necrotic phase. Simultaneously, the CEs of *L. plantarum* 0991 had no influence on cell death induction.

According to the authors’ knowledge, the effects of PFM and CEs on PS externalisation and membrane permeabilisation have not been investigated before. LAB can induce apoptosis in cancer cells, which is an important mechanism for inhibiting tumour progression. Therefore, many research studies have confirmed the ability of PFM and CEs to induce apoptosis in cancer cells such as SW-480, Caco-2, HT-29, AGS, HeLa, MCF-7, Hep-G2, PC-3 [14,17,18,19,34,46,49]. CFS and conditioned media of *L. plantarum* NCIMB 5221, *L. acidophilus* ATCC 314, and *L. rhamnosus* ATCC 53103 decreased the ATP level and induced apoptosis in SW-480 and Caco-2 cell lines in the dose- and time-dependent manner [18]. *L. acidophilus* and *L. delbrueckii* CEs showed the ability to induce apoptosis in HT-29 cells at concentrations of 2 and 4 mg/mL, respectively [32]. The above two studies investigated apoptosis via measurement of caspase 3 and/or 7 levels, suggesting that it occurs on the mitochondrial signalling pathway, as in our research. Additionally, Wan et al. [41] detected apoptosis in colon cancer SW620 cells induced by supernatants of *L. delbrueckii* via a caspase 3-dependent pathway. Altonsy et al. [51] observed the proapoptotic activity of live cells of *L. rhamnosus* GG and *B. animalis* ssp. *lactis* Bb-12, which induced apoptosis of Caco-2 cells via the mitochondrial pathway by activation of caspase-3 and -9. Furthermore, intact live cells of *L. rhamnosus* GG and *L. paracasei* IMPC2.1 and HK cells could induce apoptosis of gastric HGC-27 and colon DLD-1 cancer cell lines [16]. PFM and CEs can also induce necrosis in cells [17,43]. Soltan Dallal [43] observed that CEs induced the number of necrotic Caco-2 cells more than PFM, concluding that LAB can induce necrosis via direct effects, not by secreted metabolites.

### 3.5. Effects of PFM and CE Treatment on Cell Monolayer and Cell Morphology

**Figure 7:**
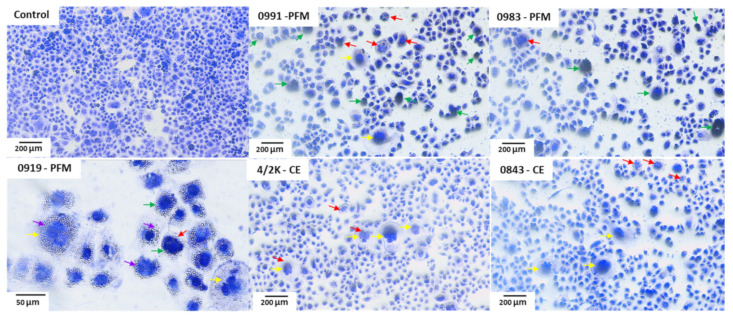
Images demonstrating morphology of Caco-2 cells after 24 h exposure to 10% (*v/v*) post-fermentation media (PFM) or cell extracts (CEs) of selected LAB stained with crystal violet. Observations under inverted microscope (20× and 40× objectives) Nikon Ts2 with EMBOSS contrast (Nikon, Tokyo, Japan). Arrows: red (chromatin fragmentation); green (chromatin condensation); yellow (cell swelling); violet (vacuolisation of the cytoplasm).

Staining of cells and their observation in a microscope is a useful tool for determining morphological changes in cells/cell membrane and changes in nuclear chromatin condensation and the cytotoxic effect of various chemical agents. Cell monolayers and cell morphology after 24 h exposition to 10% (*v/v*) PFM and CEs of LAB are presented in Figure 7. Changes in the morphology of treated cells were observed after staining with crystal violet. In the untreated control, cells were regular in shape with clearly outlined cytoplasm and cell nucleus, and they formed a confluent monolayer. A reduction in the Caco-2 cells density in monolayer, as well as semi-detached and floating cells, were observed. In all treated cells, the number of cells per visual field was fewer than in the negative control, and the monolayer lost its confluency. Chromatin fragmentation and condensation, cell swelling, and cytoplasmic vacuolisation were observed.

**Figure 8:**
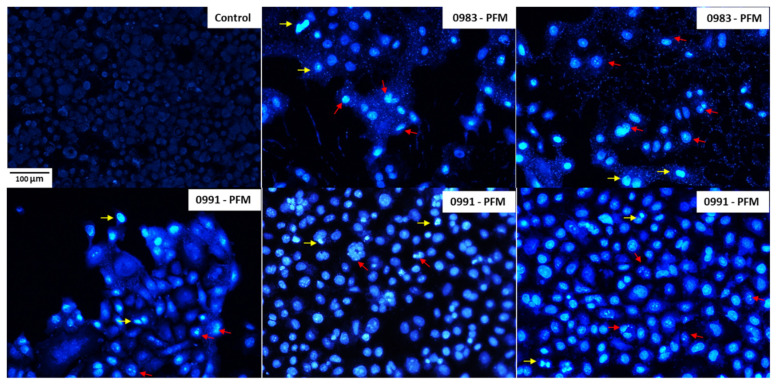
4′,6-Diamidino-2-phenylindole (DAPI) staining of Caco-2 cells exposed to post-fermentation media (PFM) of *L. plantarum* 0991 and *L. brevis* 0983 observed under a fluorescent microscope (Nikon Eclipse Ci H600L, Japan), 20× objective. Arrows: red (chromatin fragmentation); yellow (chromatin condensation).

Nuclear abnormalities were visualised after staining with DAPI (Figure 8). In Figure 8, sample microphotographs of PFM of *L. plantarum* 0991 and *L. brevis* 0983 are presented, as their ability to induce apoptosis was demonstrated in caspases 3/7 and 9 tests. Intact cells were rhomboidal, and the nuclei were homogenously stained, producing light fluorescence. Chromatin fragmentation and condensation, which are considered the main symptoms of apoptosis, were also observed in many cells after exposition to PFM. This experiment confirmed the ability of investigated PFM to induce apoptosis in Caco-2 cells. Several studies followed DAPI or Hoechst staining of cells, along with other assays for apoptosis detection [14,17,19,32,45], and they observed similar changes in nuclear morphology as achieved in our investigation.

### 3.6. SCFA and LA Profiles in PFM and Cytotoxic Activity of Pure Acids

**Figure 9:**
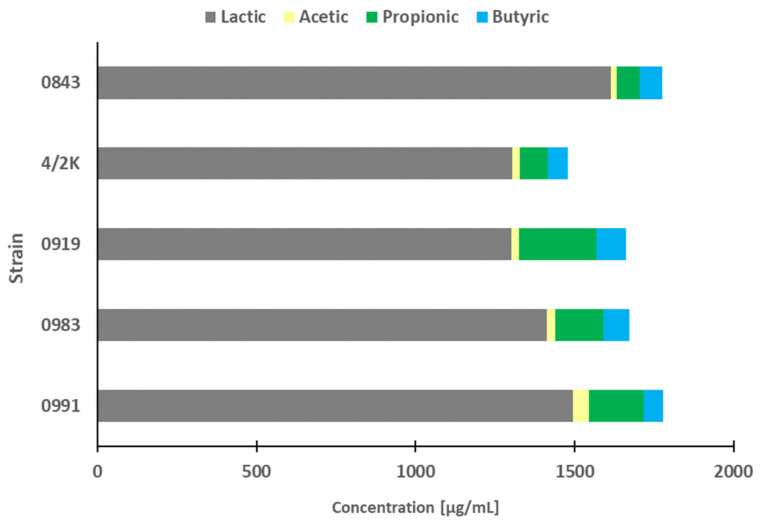
Lactic acid (LA) and short-chain fatty acids (SCFA) profiles in post-fermentation media (PFM) of selected lactic acid bacteria (LAB) with the strongest antiproliferative activity.

In our study, in order to disqualify the effect of the acids, the pH of PFM was set to neutral. As previously mentioned, the physiological acidic pH of PFM is only partly involved in the antiproliferative effect against cancer cells, and it is stronger when live LAB cells are co-incubated with cancer cells because they can decrease the pH more effectively than PFM alone [10]. Thus, the ability of selected LAB to produce LA and SCFAs was evaluated by measurement of their levels in the PFM. LA and SCFA production by LAB strains is a very important future for candidates of ‘probiotic’ status. The profiles of LA and SCFAs produced by LAB are presented in Figure 9. High amounts of butyric and propionic acids were produced by strain *L. plantarum* 0991 (i.e., 61.08 ± 9.84 and 173.36 ± 0.44 µg/mL, respectively), followed by the strain *L. brevis* 0983 (i.e., 81.02 ± 4.69 and 152.63 ± 0.36 µg/mL, respectively). Specifically, the PFM of these strains in our study induced apoptosis in Caco-2 cells. However, the most efficient producer of butyric and propionic acids, as well as in total, was strain *L. casei* 0919, which produced 359.85 µg/mL of total SCFAs, including 92.81 ± 2.90 µg/mL of butyric and 242.58 ± 1.12 µg/mL of propionic acids.

Additionally, the cytotoxic dose-dependent effects of pure LA and SCFAs on Caco-2 cells were measured, and their IC_50_ values were estimated (dose-dependent curves are not presented). The antiproliferative activity of LA and SCFAs presented as follows: butyric (IC_50_ 0.04%) > propionic (IC_50_ 0.1%) > acetic (IC_50_ 0.2%) > lactic (IC_50_ 0.4%). Based on a comparison of the IC_50_ values of pure acids with the IC_50_ values of the PFM of our selected LAB (Table 3), pure acids show a much stronger antiproliferative effect against Caco-2 cells than PFM.

Of all SCFAs in the human colon acetate, propionate and butyrate are the most abundant (≥95%), and they are present usually in an approximate molar ratio of 60:20:20, but it depends on many factors such as type of diet, age, and chronic diseases [52]. The concentrations of acetic, propionic, and butyric acids follow the formula proposed by Topping et al. [53], acetate > propionate = butyrate, but the molar ratio of SCFAs is strain-specific. Anticancer effects of SCFAs, especially butyrate, is well documented; its proper concentrations reduce proliferation and induce programmed cell death in CRC and other cancer cells [54]. Propionic acid can induce ROS generation, MMP depletion, and cell death (autophagy) in HeLa cells [55].

## 4. Conclusions

In conclusion, our study found that the antiproliferative activity of LAB against cell lines is specific for a strain rather than a species or genus. Both PFM and CEs showed inhibitory effects against cancerous (Caco-2 and HeLa) and normal (IEC-6) cells, the effect being weaker against normal cells (IEC-6) in the presence of lower concentrations of PFM and CEs, i.e., 1% and 5%. In general, PFM are more inhibitory than CEs, but this is also a strain-specific feature. PFM and CEs of some selected strains generate oxidative stress in Caco-2 cells by inducing hydrogen peroxide production and induction of ROS. PFM of two strains—*L. plantarum* 0991 and *L. brevis* 0983—induced apoptosis, confirmed by detection of activity of caspases 3/7 and 9, which suggests the mitochondrial signalling pathway of apoptosis, especially in the presence of lower concentrations at higher doses, may be late apoptosis or necrosis. Both strains are good material for further research; perhaps in the future, these strains and/or their metabolites (postbiotics) could be applied as food additives with anticancer activity.

Food ingredients may play key roles in the aetiology of CRC. They can both contribute to its formation and prevention. The latter may include probiotics and beneficial postbiotics produced by them. They can be used in the prophylaxis of CRC and its early treatment by inhibiting carcinogenesis. Therefore, to produce probiotic/postbiotic preparations, it is worth selecting LAB strains and their metabolites with confirmed anticancer properties. However, in order to apply them in biotechnology and the pharmaceutical industry, many studies confirming their pro-health benefits for the human body, and above all clinical trials, must first be conducted.

## Figures and Tables

**Table 1:** Antiproliferative and cytotoxic activity of post-fermentation media (PFM) and cell extracts (CEs) as determined by 3-(4,5-dimethylthiazol-2-yl)-2,5-diphenyltetrazolium bromide (MTT) assay in the Caco-2 cell line after 48 h exposition. Each data point represents the mean ± SD, *n* ≥ 4. * Result significantly different from untreated control cells, *p* ≤ 0.05. The strains with the strongest antiproliferative activity selected for further studies are bolded.

Strain	Cytotoxicity (%) ± SD							
	PFM Concentration (%)				CE Concentration (%)			
	1	5	10	20	1	5	10	20
*Lactiplantibacillus plantarum* 0981	3.9 ± 2.4	3.0 ± 2.5	23.4 ± 0.9 *	21.9 ± 1.6 *	3.8 ± 3.2	10.4 ± 1.9	27.0 ± 3.3 *	36.4 ± 3.9 *
*Lactiplantibacillus plantarum* 0982	−2.6 ± 1.9	0.6 ± 1.3	1.2 ± 2.3	14.2 ± 3.5	13.3 ± 3.4	22.7 ± 3.3 *	21.0 ± 1.7 *	27.6 ± 1.5 *
*Lactiplantibacillus plantarum* 0989	1.9 ± 3.2	7.1 ± 2.6	10.7 ± 0.0	2.6 ± 0.8	0.3 ± 2.1	8.6 ± 3.0	30.5 ± 4.8 *	35.4 ± 1.7 *
*Lactiplantibacillus plantarum* 0990	−12.8 ± 2.1	2.0 ± 5.3	9.3 ± 2.9	25.2 ± 2.5 *	10.0 ± 1.6	17.8 ± 2.5 *	27.3 ± 3.3 *	37.8 ± 4.0 *
***Lactiplantibacillus plantarum* 0991**	**9.3 ± 0.1**	16.7 ± 0.2 *	23.5 ± 0.7 *	61.5 ± 0.2 *	**14.2 ± 2.7**	26.6 ± 2.4 *	36.5 ± 0.5 *	45.9 ± 0.6 *
*Lactiplantibacillus plantarum* 0995	9.8 ± 0.3	44.1 ± 0.5 *	56.6 ± 0.9 *	63.2 ± 0.3 *	17.1 ± 0.0 *	23.7 ± 1.1 *	24.2 ± 0.8 *	25.7 ± 1.9 *
*Lactiplantibacillus plantarum* 0996	4.1 ± 1.1	64.0 ± 0.6 *	60.6 ± 0.4 *	64.0 ± 0.5 *	1.7 ± 2.7	20.0 ± 0.7 *	17.4 ± 0.7 *	26.7 ± 2.3 *
***Levilactobacillus brevis* 0983**	34.6 ± 0.6 *	35.6 ± 0.8 *	55.8 ± 0.4 *	71.2 ± 0.1 *	**4.0 ± 2.5**	29.0 ± 3.4 *	38.5 ± 4.0 *	36.5 ± 0.5 *
*Levilactobacillus brevis* 0984	12.5 ± 0.4	11.8 ± 0.6	12.0 ± 0.8	19.4 ± 1.0 *	2.6 ± 3.5	1.8 ± 0.2	28.3 ± 2.3 *	28.0 ± 2.3 *
*Levilactobacillus brevis* 0912	−16.7 ± 2.1	32.6 ± 1.5 *	70.1 ± 1.0 *	77.8 ± 0.8 *	18.9 ± 2.6 *	17.7 ± 4.9 *	19,3 ± 1.2 *	18.9 ± 1.3 *
*Levilactobacillus brevis* 0950	2.8 ± 0.8	16.7 ± 0.1 *	12.9 ± 0.1	29.2 ± 0.2 *	−1.24 ± 2.6	11.5 ± 2.5	12.1 ± 0.5	28.9 ± 1.2 *
*Lacticaseibacillus paracasei* 0985	−0.47 ± 0.0	26.9 ± 0.9 *	35.2 ± 0.7 *	44.4 ± 0.9 *	16.1 ± 0.9 *	39.2 ± 0.8 *	39.1 ± 1.3 *	46.5 ± 3.5 *
*Lacticaseibacillus paracasei* 0913	1.5 ± 3.5	−2.3 ± 0.4	39.0 ± 2.4 *	41.0 ± 2.0 *	9.7 ± 2.0	18.7 ± 2.1 *	31.2 ± 1.9 *	28.1 ± 1.7 *
*Lacticaseibacillus paracasei* 0993	−10.7 ± 0.7	23.9 ± 0.2 *	20.9 ± 1.3 *	38.4 ± 0.3 *	16.9 ± 2.9 *	21.5 ± 2.9 *	31.2 ± 2.0 *	29.6 ± 1.7 *
*Lactobacillus delbrueckii* 0851	28.1 ± 0.4 *	35.0 ± 0.2 *	41.8 ± 0.7 *	42.6 ± 0.6 *	13.0 ± 1.1	19.9 ± 1.2 *	22.6 ± 2.2 *	35.1 ± 2.1 *
*Lactobacillus delbrueckii* 0987	−10.4 ± 2.3	2.8 ± 2.6	−3.7 ± 0.4	22.8 ± 1.4 *	17.4 ± 4.1 *	21.1 ± 3.5 *	21.8 ± 4.0 *	27.8 ± 0.7 *
*Leuconostoc mesenteroides* 0986	20.0 ± 0.5 *	21.4 ± 1.0 *	26.3 ± 0.0 *	48.4 ± 1.6 *	−0.3 ± 0.1	24.7 ± 1.7 *	30.2 ± 2.9 *	31.3 ± 0.9 *
*Leuconostoc mesenteroides* 0994	3.9 ± 3.6	14.7 ± 1.1	14.2 ± 3.0	56.8 ± 1.6 *	−1.5 ± 4.3	4.8 ± 3.0	16.1 ± 1.6 *	17.5 ± 1.7 *
*Lacticaseibacillus rhamnosus* 0900	−14.7 ± 1.2	−4.9 ± 2.1	4.3 ± 6.1	39.2 ± 2.2 *	7.2 ± 4.0	18.0 ± 3.8 *	17.2 ± 0.4 *	18.2 ± 3.0 *
*Lacticaseibacillus rhamnosus* 0902	15.8 ± 1.1 *	33.8 ± 1.1 *	33.0 ± 0.9 *	49.7 ± 1.4 *	18.1 ± 2.2 *	18.7 ± 1.8 *	19.6 ± 1.6 *	21.5 ± 3.1 *
*Lacticaseibacillus rhamnosus* 0908	6.9 ± 3.8	6.5 ± 0.9	14.8 ± 5.4	14.1 ± 3.2	−4.3 ± 0.0	11.5 ± 1.4	10.4 ± 3.7	14.5 ± 0.8
*Lacticaseibacillus rhamnosus* 0997	−13.7 ± 0.2	−9.7 ± 1.6	13.3 ± 2.9	13.7 ± 2.7	18.4 ± 1.6 *	31.2 ± 0.4 *	29.0 ± 1.2 *	29.1 ± 0.8 *
*Lacticaseibacillus rhamnosus* GG	2.0 ± 1.2	−1.2 ± 2.6	14.7 ± 2.4	33.6 ± 0.6 *	32.6 ± 1.3 *	47.1 ± 3.0 *	46.0 ± 1.4 *	58.4 ± 1.6 *
*Limosilactobacillus mucosae* 0988	−6.1 ± 2.1	15.4 ± 2.7 *	28.8 ± 1.6 *	20.1 ± 4.2 *	28.7 ± 2.0 *	31.6 ± 0.8 *	39.5 ± 2.2 *	44.2 ± 0.6 *
*Lacticaseibacillus casei* 0903	12.9 ± 1.9	27.4 ± 0.7 *	28.3 ± 0.9 *	40.0 ± 0.9 *	−5.0 ± 1.0	3.5 ± 3.9	13.9 ± 3.9	31.1 ± 2.3 *
***Lacticaseibacillus casei* 0919**	33.0 ± 0.2 *	40.4 ± 1.6 *	49.9 ± 1.5 *	64.4 ± 0.5 *	**6.1 ± 1.4**	**10.1 ± 3.5**	30.9 ± 1.0 *	39.5 ± 1.2 *
*Lacticaseibacillus casei* 0924	26.6 ± 0.4 *	22.5 ± 1.1 *	23.9 ± 0.9 *	40.8 ± 1.2 *	34.8 ± 2.6 *	41.9 ± 2.5 *	40.7 ± 3.5 *	46.7 ± 3.8 *
*Lacticaseibacillus casei* DN 114-001	13.8 ± 1.3	31.2 ± 1.0 *	29.8 ± 0.3 *	32.1 ± 1.1 *	−2.6 ± 0.0	5.6 ± 2.1	4.2 ± 2.0	3.8 ± 2.9
*Lacticaseibacillus casei* Shirota	11.0 ± 0.6	30.4 ± 2.3 *	32.7 ± 2.1 *	33.8 ± 1.4 *	4.8 ± 0.9	34.5 ± 3.1 *	36.7 ± 2.2 *	33.8 ± 2.8 *
*Lactobacillus acidophilus* 0839	7.5 ± 1.8	9.4 ± 2.3	23.0 ± 1.7 *	46.4 ± 1.2 *	15.7 ± 1.6 *	43.2 ± 1.5 *	43.1 ± 0.4 *	45.7 ± 1.7 *
*Lactobacillus acidophilus* 0925	7.7 ± 0.8	16.6 ± 1.4 *	21.4 ± 1.9 *	43.9 ± 1.6 *	6.8 ± 3.4	7.6 ± 0.9	8.2 ± 2.4	19.9 ± 1.1 *
*Lactobacillus acidophilus* 0937	23.6 ± 0.5 *	25.0 ± 1.0 *	22.8 ± 2.1 *	58.6 ± 2.1 *	2.5 ± 3.3	15.2 ± 6.8 *	16.2 ± 1.5 *	28.7 ± 2.9 *
*Lactobacillus acidophilus* 0946	4.4 ± 1.3	3.3 ± 0.9	18.7 ± 0.7 *	40.4 ± 1.6 *	−0.4 ± 1.0	25.5 ± 2.6 *	27.7 ± 2.4 *	45.4 ± 1.8 *
*Lactobacillus helveticus* Z/1	18.9 ± 2.2 *	13.0 ± 0.3	24.5 ± 0.5 *	10.7 ± 0.7	4.3 ± 2.0	24.5 ± 2.1 *	27.3 ± 0.7 *	32.8 ± 2.1 *
***Amylolactobacillus amylophilus* 0843**	41.4 ± 0.8 *	46.6 ± 0.9 *	65.5 ± 1.5 *	66.7 ± 1.4 *	31.4 ± 2.8 *	30.3 ± 0.6 *	30.5 ± 1.0 *	38.3 ± 1.4 *
*Secundilactobacillus similis* 04/2	18.8 ± 0.6 *	19.6 ± 0.9 *	40.7 ± 1.1 *	46.2 ± 1.9 *	27.4 ± 2.8 *	33.5 ± 4.5 *	44.8 ± 1.2 *	47.8 ± 2.7 *
*Lentilactobacillus diolivorans* 04/1	3.5 ± 2.4	−0.7 ± 0.2	25.1 ± 0.6 *	25.5 ± 2.3 *	2.8 ± 3.1	4.9 ± 0.2	6.5 ± 1.9	17.3 ± 1.0 *
*Pediococcus parvulus* 02/1	3.4 ± 1.9	23.2 ± 2.1 *	29.4 ± 1.8 *	42.1 ± 0.6 *	18.1 ± 1.4 *	16.7 ± 0.9 *	21.4 ± 1.8 *	25.8 ± 1.5 *
***Pediococcus parvulus* 4/2K**	**3.0 ± 0.8**	30.2 ± 0.7 *	52.6 ± 0.8 *	70.5 ± 1.0 *	29.0 ± 1.1 *	32.8 ± 2.3 *	39.4 ± 1.4 *	58.3 ± 2.0 *
*Bifidobacterium breve animalis* Bb-12	9.2 ± 0.3	45.8 ± 1.6 *	55.9 ± 1.3 *	59.7 ± 1.2 *	16.5 ± 2.3 *	24.4 ± 3.1 *	28.5 ± 1.9 *	51.3 ± 0.9 *

**Table 2:** Antiproliferative and cytotoxic activity of post-fermentation media (PFM) and cell extracts (CEs) in the HeLa and IEC-6 cell lines after 48 h exposition. Each data point represents the mean ± SD, *n* ≥ 4. * Results are significantly different from unexposed control cells, *p* ≤ 0.05.

Strain	Cytotoxicity (%) ± SD							
	PFM Concentration (%) (*v/v*)				CE Concentration (%) (*v/v*)			
	1	5	10	20	1	5	10	20
	HeLa							
*Lactiplantibacillus plantarum* 0991	10.1 ± 0.0	14.1 ± 0.9	41.4 ± 0.2 *	97.6 ± 0.2 *	−2.4 ± 1.6	0.1 ± 0.7	−2.7 ± 0.2	15.7 ± 3.5 *
*Levilactobacillus brevis* 0983	9.8 ± 0.6	22.7 ± 0.3 *	42.2 ± 0.8 *	98.9 ± 0.1 *	−3.3 ± 0.2	−1.8 ± 0.2	4.7 ± 3.7	26.5 ± 2.9 *
*Lacticaseibacillus casei* 0919	27.6 ± 0.8 *	31.5 ± 0.4 *	46.8 ± 0.5 *	98.9 ± 0.1 *	−3.3 ± 0.2	−1.7 ± 0.0	8.2 ± 1.4	13.3 ± 0.8
*Pediococcus parvulus* 4/2K	11.7 ± 1.1	28.3 ± 1.0 *	43.8 ± 0.7 *	97.9 ± 0.3 *	21.6 ± 0.2 *	21.9 ± 0.3 *	28.3 ± 1.7 *	28.6 ± 1.1 *
*Amylolactobacillus amylophilus* 0843	5.5 ± 0.4	37.9 ± 1.1 *	56.1 ± 0.7 *	99.2 ± 0.1 *	−3.8 ± 0.1	−0.5 ± 0.1	−2.0 ± 0.2	−0.1 ± 0.8
	IEC-6							
*Lactiplantibacillus plantarum* 0991	−0.7 ± 1.2	10.1 ± 1.7	78.3 ± 0.1 *	96.9 ± 0.2 *	2.6 ± 0.6	20 ± 0.1 *	25.5 ± 0.6 *	41.0 ± 0.5 *
*Levilactobacillus brevis* 0983	−2.5 ± 0.2	7.8 ± 0.7	80.0 ± 0.0 *	96.1 ± 0.2 *	−0.7 ± 1.4	20.0 ± 0.4 *	20.7 ± 0.4 *	33.5 ± 0.7 *
*Lacticaseibacillus casei* 0919	8.9 ± 0.2	19.4 ± 1.1 *	79.6 ± 0.1 *	98.3 ± 0.1 *	3.0 ± 0.4	7.9 ± 1.1	20.0 ± 0.5 *	19.0 ± 0.6 *
*Pediococcus parvulus* 4/2K	5.9 ± 1.5	16.0 ± 1.2 *	76.8 ± 0.3 *	97.3 ± 0.1 *	23.1 ± 0.1 *	20.7 ± 0.4 *	24.9 ± 0.5 *	30.8 ± 0.1 *
*Amylolactobacillus amylophilus* 0843	0.2 ± 1.0	43.9 ± 0.6 *	80.0 ± 0.0 *	99.6 ± 0.1 *	2.5 ± 1.3	6.2 ± 1.0	24.5 ± 0.2 *	37.9 ± 0.3 *

**Table 3:** IC_50_ (%) values of post-fermentation media (PFM) determined for five selected lactic acid bacteria (LAB) strains.

Strain	IC_50_ (%) (*v/v*)		
	Caco-2	HeLa	IEC-6
*Lactiplantibacillus plantarum* 0991	17.0	11.5	7.9
*Levilactobacillus brevis* 0983	8.6	11.4	7.8
*Lacticaseibacillus casei* 0919	10.1	10.6	7.1
*Pediococcus parvulus* 4/2K	8.5	11.1	7.4
*Amylolactobacillus amylophilus* 0843	5.9	9.0	5.8

**Table 4:** Hydrogen peroxide release in alive Caco-2 cells after 24 h exposition to 5% (*v/v*) post-fermentation media (PFM) and cell extracts (CEs). Each data point represents the mean ± SD, *n* ≥ 4. * Results that are significantly different from unexposed control, *p* ≤ 0.05.

Strain	H_2_O_2_ (µM)	
	PFM	CE
*Lactiplantibacillus plantarum* 0991	4.0 ± 2.4	8.8 ± 2.1 *
*Levilactobacillus brevis* 0983	7.3 ± 2.2 *	10.3 ± 1.4 *
*Lacticaseibacillus casei* 0919	6.9 ± 2.8 *	18.6 ± 2.7 *
*Pediococcus parvulus* 4/2K	2.8 ± 1.9	10.1 ± 2.3 *
*Amylolactobacillus amylophilus* 0843	17.1 ± 2.9 *	15.8 ± 2.3 *

## Data Availability

The data presented in this study are available in this article and are available from the corresponding author upon reasonable request.
